# Replication analysis of variants associated with multiple sclerosis risk

**DOI:** 10.1038/s41598-020-64432-3

**Published:** 2020-04-30

**Authors:** Mohammad Dashti, Khadijah Ateyah, Raed Alroughani, Rabeah Al-Temaimi

**Affiliations:** 1 Genetics and Bioinformatics department, Dasman Diabetes Institute, Sharq, Kuwait City, Kuwait; 2 Undergraduate medical program, Faculty of Medicine, Kuwait University, Jabriya, Kuwait; 3 Neurology Clinic, Amiri Hospital, Kuwait City, Kuwait; 4 Human Genetics Unit, Department of Pathology, Faculty of Medicine, Kuwait University, Jabriya, Kuwait

**Keywords:** Multiple sclerosis, Genetics, Biomarkers, Medical research, Risk factors

## Abstract

Multiple Sclerosis (MS) is a complex chronic neurodegenerative disorder resulting from an autoimmune reaction against myelin. So far, many genetic variants have been reported to associate with MS risk however their association is inconsistent across different populations. Here we investigated the association of the most consistently reported genetic MS risk variants in the Kuwaiti MS population in a case-control study designs. Of the 94 reported MS risk variants four variants showed MS risk association in Arabs exome analysis (*EVI5* rs11808092 p = 0.0002; *TNFRSF1A* rs1800693 p = 0.00003; *MTHFR* rs1801131 p = 0.038; and *CD58* rs1414273 p = 0.00007). Replication analysis in Kuwaiti MS cases and healthy controls confirmed *EVI5* rs11808092A (OR: 1.6, 95%CI: 1.19–2.16, p = 0.002) and *MTHFR* rs1801131G (OR: 1.79, 95%CI: 1.3–2.36, p = 0.001) as MS risk genetic factors, while *TNFRSF1A* rs1800693C had a marginal MS risk association (OR: 1.36, 95%CI: 1.04–1.78, p = 0.025) in the Kuwaiti population. *CD58* rs1414273 did not sustain risk association (p = 0.37). In conclusion, *EVI5* rs11808092A, *TNFRSF1A* rs1800693C and *MTHFR* rs1801131G are MS risk factors in the Kuwaiti population. Further investigations into their roles in MS pathogenesis and progression are merited.

## Introduction

Multiple Sclerosis (MS) is a complex, chronic, neurodegenerative autoimmune disorder that affects the central nervous system (CNS). MS manifests in the CNS through formation of inflammatory lesions in the white matter of the brain and spinal cord. The lesions represent an endpoint of repeated autoimmune attacks against endogenous myelin-associated antigens which lead to neuroaxonal degeneration, and death of myelin forming cells; the oligodendrocytes. This pathological process results in demyelinated plaques or astrocytic scars throughout the CNS perturbing neural networks^1^. It is estimated that 2,221,188 individuals are living with MS worldwide, and global MS incidence is twice as many in females than in males^2^. In Kuwait, MS prevalence has increased from 31.15 per 100,000 individual in 2009, to 104.88 per 100,000 individual in 2018 with a projected trend of further increases in the future^3,4^. MS has been recognized and described 151 years ago but its underlying cause remains unknown. However, there is mounting evidence of several environmental and genetic risk factors associating with the disease^5,6^. Environmental and genetic contributing factors to MS incidence are not exclusive but are thought to be synergistic as none of these factors fully explain causation alone, nor do they consistently associate with MS risk across different populations^7^.

Among the strongest genetic susceptibility associations with MS is the major histocompatibility complex (MHC) genes region^8^. The most significant associations were seen with polymorphisms in human leukocyte antigen (HLA) HLA-DR2 alleles, however their association was found variable across different MS populations^9,10^. More MS risk associations were found with other HLA loci such as HLA-DR3, -DR4, -DQ2, -DQ6, and -DQ8 allelic variants^11^. However, the associations found with HLA haplotypes are thought to explain only 25–60% of MS genetics. A large number of linkage analyses, genome wide association studies (GWAS) and candidate gene studies have been conducted to discover the missing genetics of MS. Association studies from Kuwait reporting MS genetic risk factors are sparse, and include positive association of polymorphisms in HLA class-II (DR4, DQ6, DQ7 and DQ8) genes^12^, nitric oxide synthase (NOS) genes^13^, vitamin D receptor gene^14^, and leptin gene with MS risk^15^. However, currently there are approximately more than 200 MS genetic risk factors reported so far and replication studies from Kuwait are lacking^16^. Interestingly, the ~200 MS genetic risk factors include different susceptibility markers and modifier genes with inconsistent associations across different MS populations emphasizing ethnic, geo-epidemiological, and environmental factors associating with MS risk^17^. To confirm the association of reported MS risk genetic factors, replication studies should be conducted across different MS populations to understand the influence of ethnic and geo-epidemiological factors on MS risk. Here, we report our findings from a replication study investigating non-HLA reported MS risk variants in a sampled Kuwaiti MS population. Our objective was to assess their association in a semi-ethnically homogeneous Kuwaiti cohort as these genetic risk factors were identified from multi-ethnic MS populations.

## Results

### Reported MS variants in arab exomes

The demographics and clinical characteristics of Kuwaiti MS patients and healthy Arab controls included in the exome analysis are shown in Table 1. Of the 96 selected variants 87 (90.6%) had variable detection frequency in SureSelect V5 library only, and 9 (10.4%) were not covered by our exome library. The final list of reported MS variants that had consistent detection across all exomes included 18 (20.7%) variants of acceptable detection frequencies across the two cohorts (Supplementary Table 1). Four variants had statistically significant different allele frequencies in MS exomes compared to healthy control exomes (Table 2). These variants included; Ecotropic Viral Integration Site 5 (*EVI5*) rs11808092 (OR: 2.04, 95%CI: 1.4–2.9), TNF Receptor Superfamily Member 1 A (*TNFRSF1A*) rs1800693 (OR: 2.01, 95%CI: 1.45–2.75), Lymphocyte Function-Associated Antigen 3 (*CD58*) rs1414273 (OR: 2.2, 95%CI: 1.5–3.2), and a nominally significant and not clearly robust Methylenetetrahydrofolate Reductase (*MTHFR*) variant rs1801131 (OR: 1.4, 95%CI: 1.02–1.9). However, genotype frequencies were significantly different among the two cohorts for only three variants; *TNFRSF1A* rs1800693 (p = 0.0001), *MTHFR* rs1801131 (p = 0.024), and *CD58* rs1414273 (p = 0.0001). Since these variants showed risk association in Kuwaiti MS patients when compared to Arab healthy controls we further investigated their association with MS in an exclusively Kuwaiti nationality case-control population sample.

**Table 1:** Demographics and clinical characteristics of Kuwaiti MS (n = 113) and healthy control Arabs (n = 460) included in the investigative exome analysis.

Criteria	MS patients (n = 113)	Healthy Arab controls (n = 460)
Sex (%)		
Male	40 (35.4)	229 (49.8)
Female	73 (64.6)	231 (50.2)
Age in years (Mean ±SD^a^)	32.9 ± 9.4	40.7 ± 12.6
MS type^b^ (%)		—
RRMS	16 (14.2)	
SPMS	93 (82.3)	
PPMS	4 (3.5)	
Disease duration in years (Mean ±SD)	6.77 ± 5.73	—
EDSS^c^ (Median, IQR^d^)	2 (1.5–3.5)	—

^a^SD: Standard deviation; ^b^RRMS: Relapsing-remitting MS, SPMS: Secondary progressive MS, PPMS: Primary progressive MS; ^c^EDSS: Expanded disability status scale; ^d^IQR: Interquartile range.

**Table 2:** Statistically significant exome analysis results of reported variants associated with MS risk in this study’s case-control exome cohorts.

Gene	SNP	Allele frequency in Kuwaiti MS cases (%)	Allele frequency in Arab healthy controls (%)	P-value
*CD58*	rs1414273	G: 158 (76) A: 50 (24)	G: 722 (87.4) A: 104 (12.6)	0.00007
*EVI5*	rs11808092	C: 93 (63.3) A: 54 (36.7)	C: 687 (77.9) A: 195 (22.1)	0.00024
*MTHFR*	rs1801131	T: 120 (57.1) G: 90 (42.9)	T: 574 (65.1) G: 308 (34.9)	0.038
*TNFRSF1A*	rs1800693	T: 127 (60.5) C: 83 (39.5)	T: 631 (75.5) C: 205 (24.5)	0.00002

Variant detection frequency was variable across the two exome cohorts and computed allele frequencies reflect the proportion of alleles divided by the total number of exomes in which the variant was detected.

### Replication analysis of MS variants in kuwaiti samples

The replication Kuwaiti population sample included 170 Kuwaiti MS patients and 311 healthy Kuwaitis. Replication cohorts’ demographics and clinical characteristics are shown in Table 3. Allelic and genotype frequencies for *TNFRSF1A* rs1800693, *EVI5* rs11808092, *CD58* rs1414273 and *MTHFR* rs1801131 are shown in Table 4. *TNFRSF1A* rs1800693, *EVI5* rs11808092 and *MTHFR* rs1801131 genotype frequencies in healthy Kuwaiti controls were in Hardy-Weinberg equilibrium, except for *CD58* rs1414273 for which genotype frequencies were significantly different than those expected in European populations (p < 0.0001). *TNFRSF1A* rs1800693C allele did not sustain significant association with MS risk (OR: 1.36, 95%CI: 1.04–1.78, p = 0.025) following adjustment for multiple testing. *TNFRSF1A* rs1800693 genotype distribution differed between the two cohorts (β = −0.106, 95%CI: −0.14- (−0.009), p = 0.027) after adjusting for sex and age albeit did not reach adjusted statistical significance. However, rs1800693CC+CT genotypes significantly associated with MS risk in an autosomal dominant inheritance model (OR: 1.7, 95%CI: 1.14–2.5, p = 0.008). No sex-specific MS risk or EDSS association was found for rs1800693, whereas genotype CC marginally associated with a younger age of MS onset (β = −0.169, 95%CI: −0.33 - (−0.001), p = 0.04) after adjusting for sex and disease duration.

**Table 3:** Demographics and clinical characteristics of the replication study’s Kuwaiti case-control cohorts and their allelic and genotype frequencies for the associated MS risk variants.

Criteria	Kuwaiti MS patients (n = 170)	Kuwaiti healthy controls (n = 311)
**Sex (%)**		
Male	58 (34.1)	118 (37.9)
Female	112 (65.9)	193 (62.1)
Age in years (Mean ±SD)	33 ± 8.73	30.7 ± 8.47
MS age of onset in years (Mean ±SD)	27.06 ± 7.68	
MS type (%)		—
RRMS	148 (87)	
SPMS	14 (8.2)	
PPMS	4 (2.4)	
Benign MS	4 (2.4)	
Disease duration in years (Mean ±SD)	5.6 ± 5.17	—
EDSS (Median, IQR)	1.5 (1–3)	—

**Table 4:** Replication cohorts’ allelic and genotype distributions [n (%)] of the exome analysis resultant MS risk variants.

Variant	MS cohort	Healthy controls	P-value
	(n = 170)	(n = 311)	
*CD58* rs1414273			
A/G	73 (21.5)/267 (78.5)	137 (22)/485 (78)	0.8
AA	7 (4.1)	25 (8)	0.11
AG	59 (34.7)	87 (28)	
GG	104 (61.2)	199 (64)	
*EVI5* rs11808092			
A / C	110 (32.4)/230 (67.6)	141 (22.6)/481 (77.3)	0.002
AA	20 (11.7)	15 (4.8)	0.003
AC	70 (41.2)	111 (35.7)	
CC	80 (47.1)	185 (59.5)	
*MTHFR* rs1801131			
G / T	149 (43.8)/191 (56.2)	189 (30.4)/433 (69.6)	0.00003
GG	31 (18.2)	26 (8.4)	0.0001
GT	87 (51.2)	137 (44)	
TT	52 (30.6)	148 (47.6)	
*TNFRSF1A* rs1800693			
C / T	143 (42)/197 (58)	216 (34.7)/406 (65.3)	0.025
CC	27 (15.9)	42 (13.5)	0.03
CT	89 (52.3)	132 (42.4)	
TT	54 (31.8)	137 (44.1)	

*EVI5* rs11808092A allele showed significant MS risk association in the Kuwaiti population sample (OR: 1.6, 95%CI: 1.19–2.16, p = 0.002). *EVI5* rs11808092 genotype distribution differed between the two cohorts (β = −0.195, 95%CI: −0.24- (−0.06), p = 0.001) when adjusted for age and sex. In addition, *EVI5* rs11808092 genotype association with female MS risk was stronger than in males (p = 0.007 in females vs. p = 0.042 in males). *EVI5* rs11808092 associated significantly with MS risk in both; autosomal dominant (OR: 1.65, 95%CI: 1.1–2.4, p = 0.009) and recessive (OR: 2.63, 95%CI: 1.32–5.2, p = 0.009) inheritance models. *MTHFR* rs1801131G allele significantly associated with MS risk (OR: 1.79, 95%CI: 1.3–2.36, p = 0.00003). *MTHFR* rs1801131 genotype distribution differed between the two cohorts (β = −0.23, 95%CI: −0.25- (−0.09), p = 0.0001) after adjusting for sex and age. *MTHFR* rs1801131 GG + GT strongly associated with MS risk in an autosomal dominant inheritance model (OR: 2.06, 95%CI: 1.39–3.1, p = 0.0003). Lastly, *CD58* rs1414273A allele did not show any MS risk association in the replication Kuwaiti only population sample. *CD58* rs1414273 genotypes were not significantly different with or without adjusting for sex (p = 0.69 and 0.38; respectively). *CD58* rs1414273 genotypes did not associate with MS risk in any of the inheritance models applied. None of the assessed variants associated with EDSS with or without adjusting for disease duration or/and sex.

## Discussion

There are currently more than 200 reported MS genetic risk factors stemming from linkage analysis studies, GWAS studies, functional candidate gene approaches, exome sequencing association studies, and animal model and post mortem MS brain candidate gene studies. Only a handful of these genetic factors sustained MS risk association across different MS populations, and were linked to MS pathogenesis which provided valuable potential therapeutic targets. Here, we focused on reported non-HLA MS risk variants that might be captured in exome libraries available for clinical research. We noticed that exome coverage is not consistent across samples, and different libraries have variable exome coverage as well. Four candidate genetic factors were found to have statistically significant MS risk association in the Kuwaiti MS exome cohort when compared to Arab healthy controls. However, the Kuwaiti only replication cohorts; which adjusted for possible genetic background variation/bias in the exome study, highlighted the influence of ethnicity and genetic background in false positive results of genetic studies.

*TNFRSF1A* rs1800693, *EVI5* rs11808092, and *MTHFR* rs1801131 have been reported to associate with MS risk in mostly European Caucasian populations (Supplementary Table 2). However, the effect size of these associations is variable and is relatively small depending largely on the number of samples analyzed. TNFRSF1A is a membrane-bound and soluble receptor for tumor necrosis factor-alpha (TNFα) that plays a role in cellular survival, apoptosis and inflammation in the immune and nervous systems^18^. *TNFRSF1A* rs1800693 is an intronic variant that has been shown to impact the splicing of *TNFRSF1A* mRNA resulting in a novel isoform that blocks the action of TNFα^19^. Rs1800693 association with MS risk has been shown to have consistently a small effect size in different MS populations^20^. It is possible that the marginal association of rs1800693 variant with an earlier age of MS onset seen in our study is the reason for the small effect size of this variant reported in other MS populations where younger age of MS onset cases were considerably well represented. Therefore, it is probable that due to the low representation of MS cases with younger age of MS onset in our replication MS cohort (17% with MS age of onset ≤20 years) rs1800693 association with MS risk did not reach statistical significance following adjustment for multiple testing.

*EVI5* rs11808092 variant lies in the 3′-end intron of *EVI5* gene that shares a similarity to an enhancer element and has been shown to act as a strong enhancer element on the promoter of an adjacent gene (*GFI1)* implicated in MS risk^21^. Nevertheless, *EVI5* rs11808092 as well as other variants in *EVI5* have been reported to associate with MS risk and clinical characteristics^22,23^. On the other hand, *MTHFR* rs1801131 had a nominal association with MS risk in our exome analysis, but later showed a robust association with MS risk in the Kuwaiti only cohorts. MTHFR is an enzyme involved in the conversion of 5,10-methylenetetrahydrofolate to 5-methyltetrahydrofolate and the missense variant rs1801131 (E429A) has been shown to cause decreased MTHFR enzymatic function and is associated with impaired folate metabolism and mild increases in homocysteine levels (hyperhomocysteinemia) that is reported to occur in MS patients^24,25^. Sustained elevated homocysteine levels associate with cardiovascular disturbances that are thought to predispose to MS pathogenesis and progression^26^.

Lastly, *CD58* was first identified as an MS susceptibility factor by the association of its rs12044852 variant in a study of African American MS patients and further supported by evidence of altered expression in MS^27,28^. A follow-up investigation provided two other *CD58* variants (rs2300747, rs1335532) associating with altered CD58 expression in MS^29^. Structural analysis of *CD58* coding region revealed rs1414273 to be in strong linkage disequilibrium with the *CD58* MS-associated SNP rs1335532^30^. A recent report highlighted the role of this variant in processing of the *CD58* transcript and associated microRNA in MS pathology^31^. Our exome analysis did not detect any of the other *CD58* variants except for rs1414273 and initially an association with MS risk was found in the exome analysis. However, the association was later lost in the Kuwaiti only replication analysis. It should be noted that rs1414273A is the minor allele in Kuwaitis and has a lower frequency in Kuwaitis (0.2) compared to ExAc database (0.5) and 1000 genome database (0.43) frequencies. Therefore, it is plausible that rs1414273 allelic distribution is well-retained in the majority of Kuwait’s genetic background, and rs1414273 reported linkage to rs1335532 may not be applicable to the Kuwaiti genetic background. Moreover, Qatar’s patriotization laws are very flexible with an estimated 11.6% Arab Qataris contributing to the largely immigrant 2,723,003 Qatari population^32^. Therefore, the association of rs1414273 with MS risk in our exome study was probably influenced by a non-uniform genetic background in the Qatari controls. Nevertheless, our replication study findings support the lack of rs1414273 association with MS risk in the Kuwaiti MS population but does not refute other *CD58* variants association with MS risk or CD58 altered expression in MS.

In conclusion, we have analyzed the association of a set of reported MS genetic risk variants in a semi-homogenous exome case-control study, and confirmed the results in a replication study of a homogenous genetic background. Population genetic analyses should strive to maintain genetic background uniformity to accurately ascertain population specific genetic risk susceptibilities. Our findings support the existing evidence on the roles of these genes in MS risk and pathogenesis providing further evidence into MS etiology and potential therapeutic targets.

## Methods

### Patient selection

Blood samples of 283 Kuwaiti MS patients were collected at Kuwait’s Dasman Diabetes Institute (DDI), and 311 Kuwaiti healthy control volunteers were collected by word of mouth and social networks from the Kuwaiti population. Collection criteria for MS patients included; a complete clinical MS disease profile (demographics, age of onset, type of MS, disease duration, expanded disability status scale (EDSS) score, and history of MS treatments), being a Kuwaiti citizen, and agreement to provide a 4 mL blood sample. Exclusion criteria included; having an EDSS score of 0, and a disease duration of ≤1 year. Healthy controls’ exclusion criteria included; being a non-Kuwaiti expatriate, having a family history of MS, and having a diagnosis of any complex disorder. All study protocols were approved by Kuwait’s Health Sciences Center’s Joint Committee for The Protection of Human Subjects and DDI’s ethical review committee both of which adhere to declaration of Helsinki’s Ethical Principles for Medical Research Involving Human Subjects’ guidelines. All study information and protocols were fully explained to all participants prior to procurement of their informed written consent. Of the 283 MS patients collected, 113 were prioritized as most informative for exome sequencing to include equally distributed subgroups relevant to each of the following characteristics; EDSS score, disease duration, age group, and male to female ratio of ~1:2. Whereas 56 healthy control Kuwaiti samples were age and sex matched to selected MS samples and prioritized for exome sequencing.

### DNA extraction

Blood samples were collected from all 283 MS patients, while healthy controls’ sample collection varied between blood and saliva samples. Blood samples were centrifuged at 2,500xg for 10 minutes at room temperature and three fractions were retrieved. Plasma fractions were stored at −80 °C, and white blood cells fractions (buffy coat) were subjected to DNA extraction using QIAamp DNA blood mini kit (Qiagen, CA, USA) according to manufacturer’s standard protocol. For saliva samples the same kit was used with minor modifications. For every 1 mL saliva sample 4 mL of sterile phosphate buffer saline (PBS) were added, and samples were centrifuged at 1,800 × g for 5 minutes at room temperature. Resultant pellet was suspended in 180 µl of PBS and 20 µl of kit provided proteinase K was added. The following steps where according to manufacturer’s standard kit protocol. DNA quality and quantity were assessed by a NanoDrop spectrophotometer.

### Exome sequencing

Exome sequencing of 113 Kuwaiti MS patients and 56 healthy Kuwaiti controls was performed on an Illumina HiSeq2000 platform using SureSelectXT v5 library preparation with target coverage of 50X (Illumina, CA, USA). Illumina raw paired-end reads, captured with Agilent exome library, were mapped to the human reference assembly (hg19) using Burrows wheeler aligner (BWA)^33^. Sequence Alignment Map data with average of 50X coverage were then compiled and converted to a single compressed binary file, for each strain, using SAMtools^34^. Picard software (http://picard.sourceforge.net) was used to flag PCR duplicate reads. Genome Analysis Toolkit (GATK) was used to (a) local realignment of a read that overlapped with an INDEL (b) recalibration of base quality (c) variants calling^35,36^.

### MS variant selection

A literature search was conducted using the following keywords “Multiple sclerosis genetics, Multiple sclerosis gene, multiple sclerosis polymorphism, multiple sclerosis variant, multiple sclerosis GWAS, multiple sclerosis exome sequencing, multiple sclerosis genome sequencing.” In addition, variants included in the MSgene database (MSgene.org) were also retrieved. Collected variants were filtered according to the following criteria; being a single nucleotide variant, being within intronic gene sequence, being encoded in the nuclear genome, and being an MS risk factor. A total of 96 genetic MS risk factors were selected and used for mining exome data of MS patients and healthy controls (Supplementary Table 1). Two healthy control datasets were retrieved from publically available databases that are believed to share genetic background with Kuwaitis; Qatari and middle-eastern exome sequences. Selected Qatari and Middle Eastern (non-diabetic) individuals next generation sequencing data were obtained from the national center for biotechnology information (NCBI) sequence read archive with accessions; SRP060765, SRP061943 and SRP061463. Collectively, the case-control cohorts included 113 MS exomes and 460 healthy control exomes. A Fisher exact test was carried out for each variant, and variants were selected based on p-value of significance <0.05.

### Variant genotyping in replication cohorts

DNA from a replicate MS cohort of 170 MS patients and 311 healthy controls was used to genotype polymorphisms with p-values < 0.05. Genotyping was performed using Taqman genotyping assays (Life Technologies, CA, USA) according to manufacturer’s standard protocols and analyzed using ABI 7500 Fast Real-time PCR system (Life technologies, CA, USA). Genotype allelic discrimination was determined by SDS v1.4.1 software (Applied Biosystems, CA, USA).

### Statistical analysis

Hardy-Weinberg equilibrium was assessed in the Kuwaiti healthy control cohort for the four replicated variants using allele frequencies of European ancestry as the majority of detected MS risk variants were reported from European populations. For genotype analysis Fisher’s exact-test, chi-square test, and linear and logistic regression analyses were used. All statistical analyses were performed using SPSS v.25 (IBM, NY, USA). For the replication analysis, a multiple testing Bonferroni-adjusted p-value ≤ 0.0125 was considered significant.

## Supplementary information

Supplementary Information.
