# Supplementary material for: Replication analysis of variants associated with multiple sclerosis risk
Source: Sci Rep. 2020 Apr 30;10:7327. doi: 10.1038/s41598-020-64432-3 (PMC7193640; doi:10.1038/s41598-020-64432-3)
Supplement: Supplementary file 1 — Supplementary Information. [file 41598_2020_64432_MOESM1_ESM.pdf]

Replication analysis of variants associated with multiple sclerosis risk

Mohammad Dashti, Khadijah Ateyah, Raed Alroughani, Rabeah Al-Temaimi

Mohammad Dashti[1], Khadijah Ateyah[2], Raed Alroughani[3], Rabeah Al-Temaimi[4*]

[1] Genetics and Bioinformatics department, Dasman Diabetes Institute, Sharq, Kuwait City, Kuwait.

[2] Undergraduate medical program, Faculty of Medicine, Kuwait University, Jabriya, Kuwait.

[3]Neurology Clinic, Amiri Hospital, Kuwait City, Kuwait.

[4] Human Genetics Unit, Department of Pathology, Faculty of Medicine, Kuwait University, Jabriya, Kuwait.

Supplementary Tables: 1 and 2.

Supplementary table 1. Coding and near coding variants reported to associate with MS risk in different MS populations. Highlighted in bold are variants detected in both Kuwaiti MS and healthy control Arab exomes at acceptable frequencies and their associated Fisher exact test p-values of significance.

**Gene ID**	**SNP**	**P-value**
AGAP2	rs12368653	
AHI1	rs11154801	
ALK	rs7577363	
ANKRD55	Rs6859219	
APOE	rs7412	
APOE	rs429358	
C6orf10	rs3129934	
CBLB	rs12487066	
CD226	rs763361	
CD40	rs1569723	
CD40	rs6074022	
CD58	rs1335532	
CD58	rs2300747	
CD58	**rs1414273**	0.00007
CD58	rs12044852	
CD6	rs17824933	
CDH10	rs10078091	

CDK4	rs10876994	
CLEC16A	rs11865121	
CLEC16A	rs12708716	
CYP27B1	**rs4646536**	0.09
CYP27B1	rs10877012	
CYP27B1	rs10877015	
DBC1	rs10984447	
DDX39B	rs2523506	
ERAP-1	**rs30187**	0.39
EVI5-RPL5	**rs11808092**	0.00024
EVI5-RPL5	rs10735781	
EVI5-RPL5	rs6680578	
FAM69A	rs6604026	
FAM69A	rs7536563	
FAM69A	rs11164838	
GPC5	rs9523762	
HERV-Fc1	rs391745	
HIP2	rs305124	
HLA-A	rs2523393	
HLA-DRA	rs3135388	
HLA-DRB1	rs3135388	
HLA-F-AS1	rs2523393	
HMGB1	rs12025416	

IFI30	**rs11554159**	0.93
IGHC	rs11621145	
IL10	**rs3135932**	0.78
IL23A	rs2066808	
IL23A	rs2371494	
IL23A	rs11575248	
IL23R	**rs1884444**	0.19
IL2RA	rs12722489	
IL2RA	rs2104286	
IL7R	**rs6897932**	0.44
IL7R	rs1520333	
IL7RA	rs2587156	
IRF8	rs17445836	
KANK1	rs10975200	
KIAA0350	rs6498169	
KIF1B	rs10492972	
KIF21B	rs12122721	
KIF5A	rs1678542	
KLC1	rs8702	
KLRB1	rs4763655	
LEPR	rs1137101	
MEFV	**rs3743930**	0.15
MEFV	rs104895094	

METTL-1	rs703842	
MGAT5	rs1257169	
mir-23a	rs3745453	
MTHFR	**rs1801133**	0.83
MTHFR	**rs1801131**	0.038
NCOA5	rs6074022	
NLGN1	rs13067869	
NLRP11	rs299175	
NLRP11	**rs16986626**	0.41
NR1H3	**rs2279238**	0.44
NR1H3	rs7120118	
nt13708	rs28359178	
OR8D4	**rs7942047**	0.33
PARK2	rs199657839	
PDE4B	rs1321172	
PRKCA	rs7220007	
PRKCA	rs887797	
PRKCA	rs1010544	
PRKCA	rs3890137	
PRKCA	rs2078153	
RAB38	rs1386330	
RELN	rs17157903	
RPL5	rs6604026	

SH2B3	**rs3184504**	0.11
Stat3	rs744166	
STK11	rs9282860	
TMEM39A	**rs1132200**	0.51
TNFRSF1A	rs4149584	
TNFRSF1A	**rs1800693**	0.00002
TSFM	rs6581155	
TYK2	rs34536443	
VLA-4	rs3135388	
ZC3HAV1	**rs3735007**	0.78

Supplementary table 2. Examples of evidence from different MS populations on the association of *TNFRSF1A* rs1800693, *EVI5* rs11808092, and *MTHFR* rs1801131with MS risk.

Genetic variant	Population	Reference
TNFRSF1A rs1800693	Netherlands, Switzerland, UK, USA	[1]
	Belgium, Finland, Germany, Italy, Norway, Spain, Sweden, UK, USA	[2]
	Slovakia	[3]
	Germany	[4]
EVI5 rs11808092	Spain	[5]
	New Zealand, Australia	[6]
	Meta-analysis (New Zealand, Australia, UK, USA, Spain)	[7]
MTHFR rs1801131	Germany	[8]
	Tunisia	[9]
	Turkey	[10]

References

1 De Jager, P. L. *et al.* Meta-analysis of genome scans and replication identify CD6, IRF8 and TNFRSF1A as new multiple sclerosis susceptibility loci. *Nature genetics* **41,** 776-782, doi:10.1038/ng.401 (2009).

2 International Multiple Sclerosis Genetics, C. The genetic association of variants in CD6, TNFRSF1A and IRF8 to multiple sclerosis: a multicenter case-control study. *PloS one* **6,** e18813, doi:10.1371/journal.pone.0018813 (2011).

3 Javor, J. *et al.* TNFRSF1A polymorphisms and their role in multiple sclerosis susceptibility and severity in the Slovak population. *International journal of immunogenetics*, doi:10.1111/iji.12388 (2018).

4 Hoffjan, S. *et al.* Association of TNFAIP3 and TNFRSF1A variation with multiple sclerosis in a German case-control cohort. *International journal of immunogenetics* **42,** 106-110, doi:10.1111/iji.12183 (2015).

5 Alcina, A. *et al.* Tag-SNP analysis of the GFI1-EVI5-RPL5-FAM69 risk locus for multiple sclerosis. *European journal of human genetics : EJHG* **18,** 827-831, doi:10.1038/ejhg.2009.240 (2010).

6 Australia & New Zealand Multiple Sclerosis Genetics, C. Genome-wide association study identifies new multiple sclerosis susceptibility loci on chromosomes 12 and 20. *Nature genetics* **41,** 824-828, doi:10.1038/ng.396 (2009).

7 Liu, J. *et al.* Association of EVI5 rs11808092, CD58 rs2300747, and CIITA rs3087456 polymorphisms with multiple sclerosis risk: A meta-analysis. *Meta gene* **9,** 97-103, doi:10.1016/j.mgene.2016.04.005 (2016).

8 Klotz, L. *et al.* The variant methylenetetrahydrofolate reductase c.1298A>C (p.E429A) is associated with multiple sclerosis in a German case-control study. *Neuroscience letters* **468,** 183-185, doi:10.1016/j.neulet.2009.10.057 (2010).

9 Fekih Mrissa, N. *et al.* Association of methylenetetrahydrofolate reductase A1298C polymorphism but not of C677T with multiple sclerosis in Tunisian patients. *Clinical neurology and neurosurgery* **115,** 1657-1660, doi:10.1016/j.clineuro.2013.02.025 (2013).

10 Cakina, S., Ocak, O., Ozkan, A., Yucel, S. & Ozisik Karaman, H. I. Relationship between genetic polymorphisms MTHFR (C677T, A1298C), MTR (A2756G) and MTRR (A66G) genes and multiple sclerosis: a case-control study. *Folia neuropathologica* **57,** 36-40, doi:10.5114/fn.2019.83829 (2019).
